# PE-MCAT: Leveraging Image Sensor Fusion and Adaptive Thresholds for Semi-Supervised 3D Object Detection

**DOI:** 10.3390/s24216940

**Published:** 2024-10-29

**Authors:** Bohao Li, Shaojing Song, Luxia Ai

**Affiliations:** 1 School of Intelligent Manufacturing and Control Engineering, Shanghai Polytechnic University, Shanghai 201209, China; 20221513056@stu.sspu.edu.cn; 2 School of Computer and Information Engineering, Shanghai Polytechnic University, Shanghai 201209, China; 3 School of Artificial Intelligence and Automation, Huazhong University of Science and Technology, Wuhan 430074, China; luxiaai@hust.edu.cn

**Keywords:** semi-supervised learning, pseudo-label, multi-feature fusion, point enrichment, adaptive threshold

## Abstract

Existing 3D object detection frameworks in sensor-based applications heavily rely on large-scale annotated data to achieve optimal performance. However, obtaining such annotations from sensor data—like LiDAR or image sensors—is both time-consuming and costly. Semi-supervised learning offers an efficient solution to this challenge and holds significant potential for sensor-driven artificial intelligence (AI) applications. While it reduces the need for labeled data, semi-supervised learning still depends on a small amount of labeled samples for training. In the initial stages, relying on such limited samples can adversely affect the effective training of student–teacher networks. In this paper, we propose PE-MCAT, a semi-supervised 3D object detection method that generates high-precision pseudo-labels. First, to address the challenges of insufficient local feature capture and poor robustness in point cloud data, we introduce a point enrichment module. This module incorporates information from image sensors and combines multiple feature fusion methods of local and self-features to directly enhance the quality of point clouds and pseudo-labels, compensating for the limitations posed by using only a few labeled samples. Second, we explore the relationship between the teacher network and the pseudo-labels it generates. We propose a multi-class adaptive threshold strategy to initially filter and create a high-quality pseudo-label set. Furthermore, a joint variable threshold strategy is introduced to refine this set further, enhancing the selection of superior pseudo-labels.Extensive experiments demonstrate that PE-MCAT consistently outperforms recent state-of-the-art methods across different datasets. Specifically, on the KITTI dataset and using only 2% of labeled samples, our method improved the mean Average Precision (mAP) by 0.7% for cars, 3.7% for pedestrians, and 3.0% for cyclists.

## 1. Introduction

Three-dimensional object detection has always been a core research task in the field of autonomous driving. Existing fully supervised learning methods based on deep learning [1,2,3,4,5,6] mostly rely on rich and high-quality annotations as training data. This reliance inevitably leads to significant time consumption and cost. To address this issue, semi-supervised learning (SSL) has garnered widespread attention. Its core idea is to improve the model’s generalization ability by leveraging a limited amount of labeled data along with a large amount of unlabeled data.

The application of semi-supervised 3D object detection algorithms is not limited to the field of autonomous driving but also shows great potential in areas such as robot navigation, ref. [7] drone surveillance, augmented reality (AR), and industrial automation. These scenarios have a high demand for 3D information, and semi-supervised algorithms can effectively utilize large amounts of unlabeled data, thereby improving detection accuracy and reducing the cost of manual labeling.

Most semi-supervised 3D object detection methods [8,9,10,11,12] adopt the teacher–student model strategy, which has become a mainstream research framework. Specifically, in this framework, the teacher model uses weakly augmented input scenes to generate pseudo-labels. Meanwhile, the student model uses the same scenes with strong augmentation as input and is trained using the pseudo-labels generated by the teacher model. The quality of these pseudo-labels is crucial to the detection performance of the student network.

Because semi-supervised learning requires only a small number of labeled samples, relying solely on limited samples during the early stages of training undoubtedly affects the effective training of the teacher–student network. Currently, no semi-supervised 3D object detection method has been proposed to address this issue. To overcome this challenge, we introduce a Point Augmentation Module, which directly enhances the original point cloud data. Through the Point Augmentation Module, the diversity and robustness of the original point cloud data are significantly improved. This not only enriches the expressive capacity of the training data but also effectively enhances the quality of the generated pseudo-labels.

**Figure 1:**
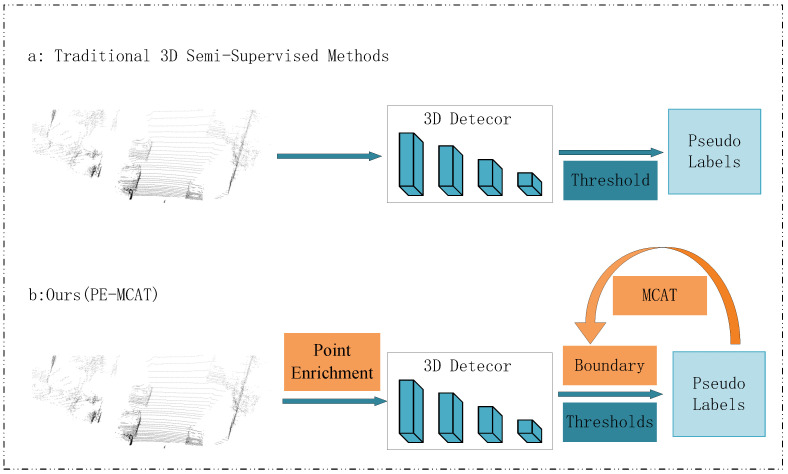
In the strategy for generating pseudo-labels, we compared our PE-MCAT method with previous methods. (**a**) Previous methods directly processed input data through a teacher network to initially generate pseudo-labels. Then, used a fixed threshold to select high-quality pseudo-labels. (**b**) The method proposed in PE-MCAT first processes the input data through point enrichment. Next, the enhanced data is input into the teacher network to initially generate pseudo-labels. Multi-class adaptive threshold (MCAT) is then applied to determine a more precise dynamic threshold. This threshold is used to filter the pseudo-labels.

With the application of the Point Augmentation Module, the quality of the generated pseudo-labels has been significantly improved. However, high-quality pseudo-labels require more precise threshold values for filtering and decomposition. Moreover, in terms of pseudo-label generation, using a fixed score threshold to obtain pseudo-labels makes it difficult to ensure their quality. If the threshold is too low, many low-quality pseudo-labels will be used to guide the student model’s training; if the threshold is too high, the number of pseudo-labels will be too few, potentially missing real objects and thus limiting the generalization ability of the student model. Although some dynamic threshold generation methods have been proposed, most of them directly use complex and inefficient pseudo-labels generated by the teacher network. The threshold value is a key parameter used to distinguish between high-confidence and low-confidence pseudo-labels. Its definition and origin are based not only on the characteristics of the augmented data but also on the improved detection capabilities of the teacher–student network during the training process. Specifically, as training progresses, the detection capabilities of the teacher–student network gradually enhance, allowing for more accurate recognition and classification of pseudo-labels. Therefore, the threshold value needs to be dynamically adjusted to adapt to the improvement in the network’s detection capabilities, thereby ensuring the accuracy and reliability of the pseudo-labels. Figure 1 illustrates the differences between the proposed method and previous works in the generation of pseudo-labels.

Specifically, the related works of PE-MCAT are as follows:

### 1.1. 3D Object Detection

Three-dimensional object detection is a key technology in fields such as autonomous driving and robotic vision, aiming to identify and locate objects from 3D point cloud data. Methods for 3D object detection can be broadly classified into point-based methods [1,4,5,6,13,14] and voxel-based methods [2,3,15,16,17,18,19].

Point-based methods directly process raw point cloud data, with the advantage of preserving detailed geometric information. The authors of PointNet [13] proposed a method to learn global features from point clouds for tasks such as classification and segmentation, while the authors of PointNet++ [14] extend this approach by introducing local feature learning to capture finer structural details. PointRCNN [1], introduced by its authors, generates a bounding box for each point and refines these proposal boxes using Non-Maximum Suppression (NMS) in a second stage.

Voxel-based methods, on the other hand, convert point cloud data into voxel grids, where 3D convolutional neural networks are applied to efficiently learn spatial information. The authors of Voxel-RCNN [2] argue that precise point-level information is not always necessary; they showed that a voxel representation, even with some loss of detail, can still achieve satisfactory recognition performance. To enhance feature extraction, Voxel-RCNN introduces Voxel ROI Pooling. Similarly, the authors of PV-RCNN [3] leverage the strengths of voxelized representation and sparse convolution by encoding multi-scale voxel features within a point cloud frame and mapping these features to key points, thus improving detection accuracy.

### 1.2. Multi-Modal Fusion

Multi-modal fusion is typically divided into data layer fusion, feature layer fusion, and object layer fusion. Data layer fusion, also known as early fusion, integrates image information with point cloud data [20,21,22,23,24,25]. The authors of PointPainting [20] proposed a method that directly enhances point clouds using images by merging image semantic segmentation results [26,27,28,29] with the original point cloud. In PointAugmenting [21], the authors use features obtained from object detection networks to achieve cross-modal data augmentation, thereby enhancing the point cloud. PointFusion [22], proposed by its authors, employs two feature extractors for each modality and performs dense fusion of the extracted features, outputting a score for each point and its predicted 3D bounding box. The authors of FusionPainting [23] combines semantic information from images and point clouds using segmentation networks and adaptively fuses the two segmentation results through an attention-based semantic fusion module.

### 1.3. Semi-Supervised 3D Object Detection

In recent years, inspired by semi-supervised 2D object detection [8,9,10,11,12], many excellent results have emerged in the field of semi-supervised 3D object detection [30,31,32,33,34]. The authors of SESS [8] proposed three consistency losses to enforce consistency between the 3D proposals from the teacher and student networks. The authors of 3DIoUmatch [9] introduced Intersection over Union (IoU) estimation as a confidence measure for localization and used 3D IoU as the localization metric. The authors also proposed the Lower-Half Suppression (LHS) module to filter out poorly localized pseudo-labels. In DetMatch [10], the authors were the first to introduce image information into the 3D semi-supervised detection framework, using Red, Green, Blue (RGB) image data to correct incorrect 3D class predictions. The authors of DDS3D [11] removed the need for NMS, allowing the teacher network to generate dense pseudo-labels, which were then filtered using dynamic thresholds. The authors of HSSDA [12] introduced hierarchical supervision using dynamic thresholds and an innovative data augmentation method called shuffle. All the above works employed the (Exponential Moving Average) EMA [35] strategy for updating the teacher network’s weights.

## 2. Method

First, PE-MCAT provides the following definitions and conventions for semi-supervised 3D object detection. In semi-supervised 3D object detection, the training dataset includes a small portion of labeled data ${\left\{x_{i}^{l},y_{i}^{l}\right\}}_{i=1}^{N_{l}}$ and a large amount of unlabeled data ${\left\{x_{i}^{u}\right\}}_{i=1}^{N_{u}}$. Here, $N_{l}$ and $N_{u}$ represent the number of labeled point cloud scenes and unlabeled point cloud scenes. $x_{i}^{l}$ and $y_{i}^{l}$ denote the input point cloud data and its corresponding annotation information.

### 2.1. Teacher-Student Framework

This study focuses on the point enrichment strategy, which involves multi-feature fusion of local features and self-features, as well as a generation strategy for pseudo-labels by the teacher network. To objectively demonstrate the effectiveness of these two strategies, PE-MCAT uses PV-RCNN [3] as the 3D detector, which is the same as the baseline model. PE-MCAT adopts a teacher–student framework. In this framework, both the student network and the teacher network use two identically configured PV-RCNNs [3] as 3D detectors. Specifically, weakly augmented unlabeled data are fed into the teacher network, while strongly augmented unlabeled data are fed into the student network. The pseudo-labels generated by the teacher network are then used to guide the training of the student network. During the training process, the EMA [35] strategy (1) is employed to facilitate parameter transfer from the student network to the teacher network.

(1) $$\theta_{t}^{i+1}=\alpha \cdot \theta_{t}^{i}+\left(1-\alpha \right)\cdot \theta_{s}^{i}$$

Here, α is the EMA momentum, $\theta_{t}$ are the parameters of the teacher model, $\theta_{s}$ are the parameters of the student model, and *i* represents the training step.

### 2.2. PE-MCAT Overview

**Figure 2:**
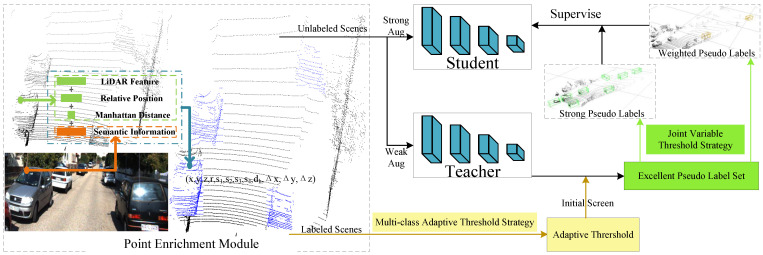
Overview of the proposed method pipeline.

The workflow of the PE-MCAT framework is shown in Figure 2. It consists of two parts. The first part is the point enrichment module based on multi-feature fusion. Initially, the semantic information of the 2D image is extracted through image semantic segmentation. Then, the calibration matrix is used to obtain the correspondence between the point cloud and the pixels. Semantic information and distance information are attached to the point cloud, achieving data-level fusion of self-features. This process extends the dimension of the point cloud from 4D to 9D. Next, dynamic spherical neighborhood sampling is employed to capture relative positional information that reflects the local features of the point cloud. This information is appended to the point cloud, which has already been fused with self-features, further extending the dimension to 12D. Finally, the dimensionally expanded point cloud is fed into the subsequent teacher–student mutual learning network.

The second part is the teacher–student mutual learning framework for semi-supervised 3D object detection. In the pre-training phase, PE-MCAT uses the labeled scenes ${\left\{x_{i}^{l},y_{i}^{l}\right\}}_{i=1}^{N_{l}}$ to train the detector in a fully supervised manner. The weights and parameters generated from pre-training are used to initialize both the teacher and student networks.During the mutual learning stage between the teacher and student networks, we first input each scene in the labeled samples and its weak augmentation into the teacher network simultaneously for prediction. The weak augmentation strategy adopted in PE-MCAT is conventional rotation and scaling. Then, we calculate the matching degree value between the predicted bounding box of each scene and its corresponding label. We separately count the detected categories to update their corresponding adaptive matching degree thresholds.

Next, we sequentially input each scene of the unlabeled samples and its corresponding weak augmentation into the teacher network. Using the newly obtained multi-class adaptive matching degree thresholds, we preliminarily screen out the scenes that meet the conditions. We add these scenes to the excellent pseudo-label set $A_{s}={\left\{x_{i}^{s},y_{i}^{s}\right\}}_{i=1}^{N_{s}}$. Then, we adopt a joint threshold strategy to obtain strong pseudo-labels and weighted pseudo-labels that specifically supervise the training of the student network.For the sets of confidence score and objectness score of the above pseudo-labels, we use clustering methods to generate two types of dynamic boundary thresholds. These thresholds match the iteratively improving detection capability of the teacher network and are denoted as $\left(\zeta_{cls},\zeta_{obj}\right)$.

Specifically, the joint variable threshold strategy uses the two dynamic boundary thresholds obtained above to filter the excellent pseudo-labels. Pseudo-labels that exceed both boundary thresholds simultaneously are considered strong pseudo-labels. Those that exceed only one of the boundary thresholds are regarded as weighted pseudo-labels. We use the strong pseudo-labels as direct labels for training the student network. For the weighted pseudo-labels, we attach variable weights to guide the student network’s training.Finally, we store the confidence score and objectness score of the strong pseudo-labels into two empty sets. Similarly, we store the corresponding scores of the weighted pseudo-labels that exceed the boundary thresholds into these sets. We then perform clustering on these sets to obtain two variable thresholds. These thresholds are used for the next iteration of the variable threshold strategy.

Finally, for the student network, a strong augmentation strategy called shuffle [12] is applied to the same scene that is input into the teacher network. This strongly augmented scene is then fed into the student network, and the strong pseudo-labels generated by the teacher network are used to guide its training. Subsequently, the teacher network is updated according to the EMA [35] strategy.

### 2.3. Point Enrichment Module

The Point Enrichment Module consists of two parts: self-feature fusion and local feature fusion. It performs multi-feature information fusion on each point in every frame of the point cloud. The first part is self-feature fusion. In the field of image semantic segmentation, DeepLabV3+ [26] is considered a high-performance model due to its accuracy, multi-scale processing capability, and generalization performance. It effectively meets the requirements of PE-MCAT for the image semantic segmentation task. PE-MCAT selects DeepLabV3+ as the baseline network for semantic segmentation to process each pixel in the image. The model ultimately generates classification confidences for the following categories: background, car, pedestrian, and cyclist.

Next, the projection module, as shown in Equation (2), uses the known extrinsic matrix $T_{lidar\text{\_}to\text{\_}camera}$ to transform the point $P(x_{lidar},y_{lidar},z_{lidar})$ in the LiDAR coordinate system into the camera coordinate system. It then uses the known intrinsic matrix *K* to map this point to the image pixel coordinates (u,v). Through the intrinsic and extrinsic matrices, each point in the point cloud is mapped to the corresponding pixel position in the 2D image, thus adding the corresponding semantic information to each point in the point cloud scene.

(2) $$\left[\begin{array}{c} u\\ v\\ 1 \end{array}\right]=K\cdot T_{lidar\text{\_}to\text{\_}camera}\cdot \left[\begin{array}{c} x_{lidar}\\ y_{lidar}\\ z_{lidar}\\ 1 \end{array}\right]$$

**Figure 3:**
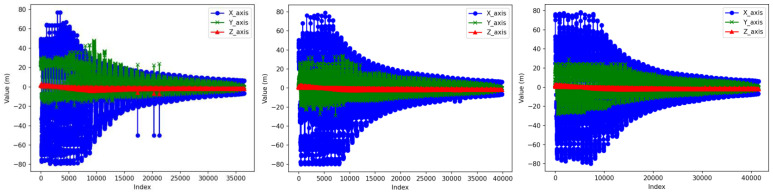
Comparison of the three-dimensional scales of the three-frame point clouds.

At the same time, we enhanced the quality of pseudo-labels by incorporating more accurate depth information. Specifically, we used the first three dimensions of each point in the point cloud to calculate its distance from the LiDAR sensor, and this distance was then appended to each point. We chose the Manhattan distance (3) as the representation of depth information, even though the Euclidean distance (4) is the most commonly used method for this purpose. However, as shown in Figure 3, in the three randomly selected point cloud scenes, there exists a subset of points. These points have relatively low features in two dimensions, while the feature in the third dimension is more prominently highlighted. If we chose the Euclidean distance to represent more accurate depth information, it would make these points very similar to the large-scale dimensional features of the point cloud. This results in an undesirable redundancy. In contrast, the Manhattan distance can intuitively introduce the ideal depth information.

(3) $$D_{\mathrm{Manhattan}}=\left|x\right|+\left|y\right|+\left|z\right|$$

(4) $$D_{\mathrm{Euclidean}}=\sqrt{x^{2}+y^{2}+z^{2}}$$

**Figure 4:**
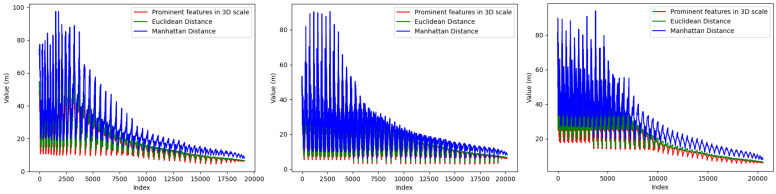
Comparison chart of the Manhattan distance, Euclidean distance, and prominent features in the three-dimensional scales of the three-frame point cloud scenes.

As shown in Figure 4, we process each point in the aforementioned three frames of point cloud scenes. For each point, we select the most prominent feature value greater than zero from its three dimensions. Since both the Manhattan distance and Euclidean distance are greater than zero, we compare the selected feature value with the Manhattan and Euclidean distances of that point. It can be seen that the Manhattan distance is more suitable for representing depth information.

The enhanced LiDAR points are transformed from the initial four dimensions (x,y,z,r) to nine dimensions ($x,y,z,r,s_{1},s_{2},s_{3},s_{4},d_{h}$). Here, $s_{1}$ represents the classification confidence for the background, $s_{2}$ for cars, $s_{3}$ for pedestrians, $s_{4}$ for cyclists, and $d_{h}$ represents the Manhattan distance. Thus, PE-MCAT achieves data-level fusion for the self-feature component.

**Figure 5:**
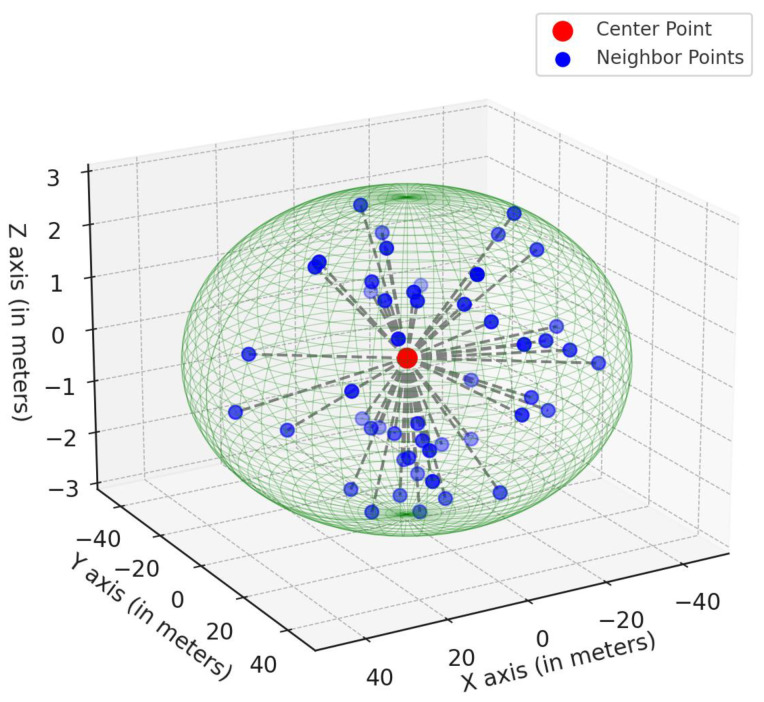
3D spherical neighborhood visualization.

The next part is local feature fusion. On the enhanced nine-dimensional point cloud, dynamic spherical neighborhood sampling is performed, as illustrated in Figure 5. For each center point $P_{c}=\left(x_{c},y_{c},z_{c}\right)$, a radius is *r* defined, and the neighboring points $P_{i}=\left(x_{i},y_{i},z_{i}\right)$ within this radius are selected. The relative position of each neighboring point with respect to the center point is then calculated, as shown in Equation (5).

(5) $$\left\{\begin{array}{c} \Delta x=x_{c}-x_{i}\\ \Delta y=y_{c}-y_{i}\\ \Delta z=z_{c}-z_{i} \end{array}\right.$$

Due to the fact that point clouds farther from the LiDAR sensor are often sparser, using a fixed neighborhood radius r may capture too many neighboring points at close distances and too few at far distances. This leads to insufficient local feature representation. Processing point clouds at all distances with a fixed radius may not fully reflect their local geometric structures. Therefore, PE-MCAT dynamically adjusts the neighborhood radius. Near the sensor, where the point cloud is dense, a smaller radius r can be used to ensure fine-grained capture of local features and to prevent the loss of local geometric details due to too many neighboring points. For sparse point clouds at greater distances, the neighborhood radius r can be increased appropriately to capture enough neighboring points, thereby ensuring the stability and completeness of local feature computation, as shown in Equation (6). Where $r_{0}$ is the base radius, α is the adjustment coefficient, and *d* is the Manhattan distance from the point cloud to the sensor.

(6) $$r\left(d\right)=r_{0}+\alpha \cdot d$$

The method adds the relative positional information obtained through dynamic spherical neighborhood sampling to the point cloud that has been enhanced in the self-feature component, expanding it from 9 dimensions to 12 dimensions, namely ($x,y,z,r,s_{1},s_{2},s_{3},s_{4},$$d_{h},\Delta x,\Delta y,\Delta z$). Here, Δx represents the horizontal local feature, Δy represents the longitudinal local feature and Δz represents the vertical local feature. Thus, PE-MCAT achieves data-level fusion of the local feature components.

### 2.4. Multi-Class Adaptive Threshold

Under the filtering of multi-class adaptive thresholds, an excellent pseudo-label set is generated. A joint variable threshold strategy is then employed to obtain strong pseudo-labels and weighted pseudo-labels that supervise the training of the student network. Algorithm 1 describes how to update the multi-class adaptive thresholds in each training epoch and the process of generating the excellent pseudo-label set based on these thresholds. Algorithm 1 utilizes the labeled sample scenes $A_{l}={\left\{x_{i}^{l},y_{i}^{l}\right\}}_{i=1}^{N_{l}}$ and their prediction pairs ${\left\{r_{i}^{l},{\tilde{r}}_{i}^{l}\right\}}_{i=1}^{N_{l}}$. In each iteration, we have c_count, which is the number of instances per class that meet the matching threshold condition. We also have all_count, representing the total number of instances per class in that iteration. Additionally, we record the adaptive threshold iou_value for each iteration. The classes being counted are denoted as Cla.

Algorithm 1 uses labeled data to generate multi-class adaptive matching thresholds in each iteration.For each scene i, there exists an original ground truth bounding box $b_{i}^{gt}$. After prediction, we obtain the bounding box $b_{i}^{l}$. We also obtain the bounding box $\tilde{b_{i}^{l}}$ from the prediction result of the weakly augmented version of the same scene. As shown in Equation (7), we calculate the matching degree, which is the IoU value between the two predicted boxes.

(7) $$S_{iou}\left(i\right)=\frac{\mathrm{Area}(r_{i}^{l}\cap {\tilde{r}}_{i}^{l})}{\mathrm{Area}(r_{i}^{l}\cup {\tilde{r}}_{i}^{l})}\circ$$

**Figure 6:**
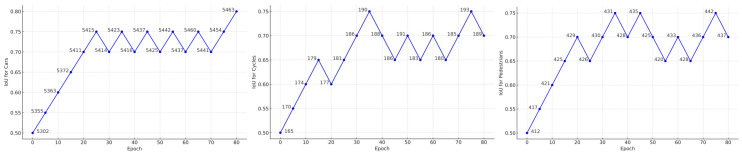
Three types of adaptive thresholding curves.

We set the IoU consistency based on $b_{i}^{l}$ and $\tilde{b_{i}^{l}}$. This allows the detection network to utilize consistency constraints, providing certain generalization capabilities on augmented scenes. To ensure some level of constraint from the first iteration, we set the default IoU threshold to 0.5, as shown in Equation (8). In the first iteration, we obtain the number of instances of each class that meet the IoU threshold. In subsequent iterations, we use the number of instances of each class obtained from the current iteration’s filtering. We compare this number with that of the same class in the previous iteration. If the positive strategy for the matching threshold of that class is satisfied, we increase its matching threshold by 0.05. Otherwise, we decrease its matching threshold by 0.05. Each class’s threshold is updated independently. As shown in Figure 6, the adaptive matching threshold variation curves for the three detected object classes are displayed. Specifically, since the pre-trained model is trained on labeled samples, the model has a certain preference and sensitivity toward these samples. Therefore, we also treat the strong pseudo-labels as labeled samples and incorporate them into the generation of multi-class adaptive thresholds.

(8) iou_value[cla][epoch=1]=0.5

**Algorithm 1** Multi-class adaptive threshold strategy
1:**Input:** labeled sample scenes $A_{l}$, pairs of predictions ${\left\{r_{i}^{l},{\tilde{r}}_{i}^{l}\right\}}_{i=1}^{N_{l}}$, the number that meets the IoU threshold in each epoch c_count, the number of epochs all_count, adaptive threshold for each epoch iou_value, the species to be tested: Cla.2:**for** eachepoch **do**3:   **for** $each\;x_{i}^{l},y_{i}^{l}$ **do**4:     $fetch\;bounding\;boxes\;b_{i}^{gt}\;from\;y_{i}^{l}$5:     $fetch\;bounding\;boxes\;b_{i}^{l}\;from\;r_{i}^{l}$6:     **for** Cla **do**7:         **for** $b_{ij}^{gt}\;in\;b_{i}^{gt}$ **do**8:            $Mx\leftarrow IoU\{b_{ij}^{gt},b_{i}^{l}\}$9:            c_count[cla][epoch]←sum(Mx>iou_value[cla][epoch−1])10:        **end for**11:        **if** $I_{da}>all\text{\_}count\left[cla\right]\left[epoch-1\right]$ **then**12:           iou_value[cla][epoch]←iou_value[cla][epoch−1]+0.0513:        **end if**14:        **if** $I_{da}<all\text{\_}count\left[cla\right]\left[epoch-1\right]$ **then**15:           iou_value[cla][epoch]←iou_value[cla][epoch−1]−0.0516:        **end if**17:     **end for**18:   **end for**19:
**end for**
20:**Output:** iou_value[cla]


Next, we sequentially input each scene of the unlabeled samples ${\left\{x_{i}^{u}\right\}}_{i=1}^{N_{u}}$ and its corresponding weak augmentation into the teacher network. Using the newly obtained multi-class adaptive matching degree thresholds from Algorithm 1, we preliminarily screen out the scenes that meet the conditions. We add these scenes to the excellent pseudo-label set $A_{s}={\left\{x_{i}^{s}\right\}}_{i=1}^{N_{s}}$, as shown in the Equation (9):

(9) $$A_{s}={\left\{\left(x_{i}^{u}\right)\right\}}_{i=1}^{N_{s}}|N_{s}=\left\{(IoU\left(b_{i}^{u},{\tilde{b}}_{i}^{u}\right)>iou\text{\_}value\left[cla\right]\right)\}$$

Algorithm 2 describes the process of obtaining strong pseudo-labels and weighted pseudo-labels using the excellent pseudo-label set with a joint variable threshold strategy. It also generates new boundary thresholds at the end. Algorithm 2 uses the excellent pseudo-label set $A_{s}={\left\{x_{i}^{s}\right\}}_{i=1}^{N_{s}}$ and its prediction pairs ${\left\{r_{i}^{s},{\tilde{r}}_{i}^{s}\right\}}_{i=1}^{N_{s}}$. Then, it creates two empty sets $S_{cls}$ and $S_{obj}$. The confidence scores and objectness scores in each set are derived from the predicted bounding boxes $b_{i}^{s}$.
**Algorithm 2** Joint variable threshold strategy1:**Input:** excellent pseudo-label set $A_{s}={\left\{x_{i}^{s}\right\}}_{i=1}^{N_{s}}$, pairs of predictions $\left\{r_{i}^{s},{\tilde{r}}_{i}^{s}\right\}$.2:$Initialize\;empty\;sets\;S_{cls},\;S_{obj}.$3:**for** eachepoch **do**4:   **for** $each\;x_{i}^{s}$ **do**5:     **for** Cla **do**6:        $Obtain\;attributes\;S_{cls}^{s},\;S_{obj}^{s},\;b_{i}^{s}\;from\;r_{i}^{s}$7:        $Obtain\;attributes\;{\tilde{S}}_{cls}^{s},\;{\tilde{S}}_{obj}^{s},\;{\tilde{b}}_{i}^{s}\;from\;{\tilde{r}}_{i}^{s}$8:        $Mx\leftarrow IoU\{b_{i}^{s},{\tilde{b}}_{i}^{s}\}$9:        **if** Mx>iou_value[Cla][epoch−1] **then**10:          $S_{cls}\leftarrow S_{cls}^{s}\;>\;\zeta_{cls}$11:          $S_{obj}\leftarrow S_{obj}^{s}\;>\;\zeta_{obj}$12:        **end if**13:     **end for**14:   **end for**15:   $\left(\zeta_{cls},\zeta_{obj}\right)\leftarrow \;K-means\left(S_{cls},S_{obj}\right)$16:**end for**17:**Output:** $\left(\zeta_{cls},\zeta_{obj}\right)$

**Figure 7:**
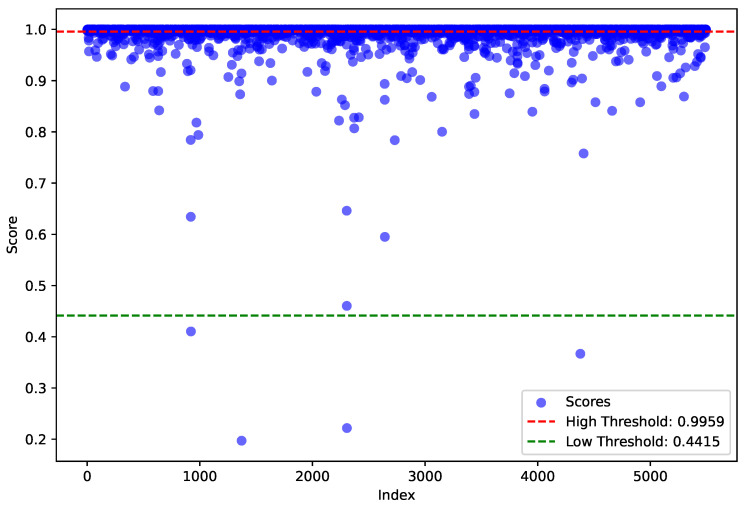
Scores and thresholds from k-means clustering for the car category.

We store the confidence scores and objectness scores of the strong pseudo-labels into the two empty sets separately. For the weighted pseudo-labels, we store the corresponding scores that exceed the boundary thresholds into the respective sets. Finally, clustering operations are performed separately on the two score sets. We group the two sets into two clusters and take the center of the high-score cluster as the corresponding threshold, Figure 7 shows the results of the high- and low-confidence threshold selection for cars. Using k-means [36], we generate two boundary thresholds $\left(\zeta_{cls},\zeta_{obj}\right)$. Using the thresholds obtained above, we classify the pseudo-labels from the teacher network’s detection of unlabeled scenes into two categories: strong pseudo-labels and weighted pseudo-labels. Strong pseudo-labels have both confidence scores and objectness scores greater than their respective thresholds (as shown in Equation (10)) and are used as direct labels for training the student network. For the remaining pseudo-labels, if only one of the scores is above its corresponding threshold, they are marked as weighted pseudo-labels (as shown in Equation (11)). These weighted pseudo-labels are assigned variable weights calculated as the product of their confidence and objectness scores, which guide the training of the student network. As the teacher network’s detection capability iteratively improves, the scores in the two sets also increase. Consequently, the boundary thresholds adapt to the enhanced detection ability of the teacher network.

(10) $$U_{i}^{\mathrm{strong}}=\left\{\begin{array}{cc} 1 & \mathrm{if}\;\left(S_{cls}\left(i\right)>\zeta_{cls}\right)\;\wedge \left(S_{obj}\left(i\right)>\zeta_{obj}\right)\\ 0 & \mathrm{otherwise} \end{array}\right.$$

(11) $$U_{i}^{\mathrm{weighted}}=\left\{\begin{array}{cc} S_{obj}\left(i\right)*S_{cls}\left(i\right) & \mathrm{if}\;\left(\left(S_{cls}\left(i\right)>\zeta_{cls}\right)\;\wedge \left(S_{obj}\left(i\right)<\zeta_{obj}\right)\right)\\ \; & \cup \left(\left(S_{cls}\left(i\right)<\zeta_{cls}\right)\;\wedge \left(S_{obj}\left(i\right)>\zeta_{obj}\right)\right)\\ 0 & \mathrm{otherwise} \end{array}\right.$$

### 2.5. Training Objective Function

PE-MCAT follows the teacher–student network paradigm, using labeled scenes with ground truth for training. Unlabeled scenes are fed to the teacher network, and the generated pseudo-labels guide the student network training.

Thus, the overall training includes both labeled scene training and unlabeled scene training. Our multi-class adaptive threshold strategy has fully utilized the labeled scenes to generate boundary thresholds. Therefore, we randomly sampled batches of data from the entire dataset for training. The final loss function consists of two parts: the loss for labeled scenes $L_{s}$ and the loss for unlabeled scenes $L_{u}$.

These are formulated as follows for labeled scenes:

(12) $$L_{s}=\sum_{i}L_{cls}\left(x_{i}^{l},y_{i}^{l}\right)+L_{reg}\left(x_{i}^{l},y_{i}^{l}\right),$$

For unlabeled scenes:

(13) $$L_{u}=\sum_{i}L_{cls}\left({\hat{x}}_{i}^{u},{\tilde{y}}_{ij}^{u}\right)+L_{reg}\left({\hat{x}}_{i}^{u},{\tilde{y}}_{ij}^{u}\right)+b_{ij}^{cls}\cdot b_{ij}^{obj}L_{cls}\left({\hat{x}}_{i}^{u},{\tilde{y}}_{ij}^{u}\right)+b_{ij}^{cls}\cdot b_{ij}^{obj}L_{reg}\left({\hat{x}}_{i}^{u},{\tilde{y}}_{ij}^{u}\right)$$

Here, $L_{cls}$ represents the classification loss. $L_{reg}$ represents the regression loss. ${\tilde{y}}_{ij}^{u}$ denotes strong pseudo-labels for unlabeled data. $b_{ij}^{cls}$ denotes predicted confidence score, $b_{ij}^{obj}$ denotes objectness reliability score. The final loss function is as follows:

(14) $$L=L_{s}+L_{u}$$

## 3. Experiments

### 3.1. Datasets

The KITTI [37] dataset is a commonly used dataset for autonomous driving, containing 7481 training samples and 7518 testing samples. In this study, the training samples are divided into a training set and a test set, comprising 3712 and 3769 scenes. Following the grouping method of 3DIouMatch [9], 1% and 2% of the training samples are separated as labeled scenes for the training phase to validate the effectiveness of the proposed method.For fair comparison, the mean Average Precision (mAP) with 40 recall positions is used as the evaluation metric. The IoU thresholds for the car, pedestrian, and cyclist categories are set to 0.7, 0.5, and 0.5.

The DAIR-V2X [38] dataset consists of a series of publicly released road vehicle cooperative datasets based on real autonomous driving scenarios. Unlike the KITTI dataset, which uses a 60-line LiDAR, DAIR-V2X employs a 40-line LiDAR on the single-vehicle side for target capture. DAIR-V2X includes 11,163 frames for training and 4464 frames for validation. PE-MCAT also separate 2% of the training samples as labeled scenes. We use the same evaluation metrics for the DAIR-V2X dataset as those used for the KITTI dataset.

Below is a formal explanation of evaluation metric used in the KITTI and DAIR-V2X dataset. mAP is a standard object detection metric that measures the accuracy of detecting and classifying objects based on the IoU between predicted and ground truth boxes.

(15) $$mAP=\frac{1}{\left|C\right|}\sum_{c\in C}AP_{c}$$

|*C*| is the total number of object classes; APc is the average precision for class *c*, calculated based on IoU thresholds. The mAP is computed by averaging AP across different IoU thresholds.

### 3.2. Implementation Details

PE-MCAT uses the base detector PV-RCNN [3] from OpenPCDet [39] for 3D detection. The proposed method (based on PV-RCNN) is trained for 80 epochs with a batch size of 6. The detector is optimized using the Adam [40] optimizer, with a maximum learning rate of 0.01. Additionally, for a fair comparison, this study adopts the same data augmentation strategy as HSSDA [12] to process unlabeled scenes input to the student network. For the data input to the teacher network, basic geometric transformations are applied. Each scene is randomly flipped along the X-axis and Y-axis with a probability of 0.5. Then, the scenes are scaled using a uniformly sampled scale factor from the range [0.91, 1.12], and rotated around the Z-axis by a random angle sampled from the range $\left[-\frac{\pi }{4},\frac{\pi }{4}\right]$. PE-MCAT uses a single 3090 Graphics Processing Unit (GPU) to conduct the experiments related to PE-MCAT.

### 3.3. Main Results

As shown in Table 1, this study trains on 1% and 2% labeled data from the KITTI [37] dataset. For fair comparison, the same detector PV-RCNN [3] is used as the baseline method, which only utilizes LiDAR data. The results in the table demonstrate that our method significantly outperforms the current state-of-the-art methods.

When using 2% labeled data, our proposed model achieves a mAP improvement of 6.1%, 21.1%, and 23.3% for cars, pedestrians and cyclists, compared to the baseline. Additionally, compared to HSSDA [12], the proposed method improves the mAP for cars, pedestrians, and cyclists by 0.8%, 3.7%, and 3.0%. Furthermore, compared to DetMatch [10], which uses both LiDAR and RGB modalities, the proposed method achieves comprehensive improvements, increasing the mAP for cars, pedestrians, and cyclists by 4.5%, 7.8%, and 4.1%.

It is noteworthy that the results obtained by using 1% labeled data for training in PE-MCAT are close to the results of HSSDA trained with 2% labeled data. When there is a small amount of labeled data for training, the point enhancement module proposed in PE-MCAT starts from the feature perspective of the labeled data itself. It employs an enhancement strategy of multi-feature fusion on point cloud data. This improves the effective feature dimensions of the labeled data themselves, ensuring the semi-supervised training effect using only a small amount of labeled data. Furthermore, the MCAT strategy proposed greatly improves the quality of pseudo-labels filtered when only a small amount of labeled data are used.

Taking everything into account, introducing a new method of multi-feature fusion and a multi-class adaptive threshold strategy into the semi-supervised field is feasible. It should be noted that for the mAP of cars, the results using 2% labeled data show that PE-MCAT only improves by 0.8% over HSSDA. This is because current methods have already achieved satisfactory results in detecting the car category during the pre-training stage. Therefore, it is difficult to improve this result using pseudo-labels under the semi-supervised framework.

As shown in Table 2, we trained on the DAIR-V2X [38] dataset using 2% labeled data. For a fair comparison, we used the same detector, PV-RCNN, as the baseline method that uses only the LiDAR single modality. The table shows that our results significantly outperform the current state-of-the-art methods. The proposed model achieves a higher mAP for cars, pedestrians, and cyclists, with improvements of 7.55%, 24.49%, and 18.22% over the PV-RCNN, respectively. Additionally, compared to HSSDA, PE-MCAT improves the mAP for pedestrians and cyclists by 12.15% and 14.25%, respectively.

### 3.4. Ablation Study

PE-MCAT conducts a series of ablation studies using labeled data to analyze the impact of the proposed strategies on the proposed method. All experiments are based on the PV-RCNN [3] detector with the 2% KITTI [37] split. The ablation results for the point enrichment(PE) module based on multi-feature fusion and the multi-class adaptive threshold strategy (MCAT) were summarized separately.

#### 3.4.1. Effect of Point Enrichment Module

From Table 3, it can be seen that the multi-feature fusion in the point enrichment module significantly improves detection performance. First, with only four-dimensional features, the model achieves a baseline performance with an mAP of 68.6%. This indicates that basic coordinate and intensity information can support the model in completing fundamental object detection tasks, but there may be limitations in complex scenes or small object detection. By adding semantic information on top of this, the detection accuracy of all categories improves, as semantic information provides prior knowledge about the category to which a point belongs, helping the model distinguish between different object categories more accurately. Furthermore, incorporating Manhattan distance as depth information enhances the model’s depth perception, making distance estimation more accurate. Finally, after fusing relative position information, the model reaches its optimal performance. The inclusion of relative position information helps capture local features, making the model more robust in the presence of occlusions or complex backgrounds, allowing it to more accurately separate objects from the background.

As shown in Table 4, on the 2% KITTI dataset, we conducted experiments on both the baseline model and the method using only the MCAT strategy, applying the point enrichment (PE) module with multi-feature fusion. The results show that the proposed point enrichment module achieved significant performance improvements in both algorithms. Under the same feature dimension conditions, PE-MCAT outperformed HSSDA, further validating the effectiveness of the proposed MCAT strategy. For the teacher network, as its detection capability improves during training, the MCAT strategy is needed to generate threshold values that match its detection capability, allowing for the more accurate selection of pseudo-labels to guide the training of the student network.

#### 3.4.2. Effect of Multi-Class Adaptive Threshold Module

To ensure a better comparison, we extensively searched for the optimal fixed matching thresholds for each category and tested various fixed matching threshold strategies as benchmarks. As shown in Table 5, the multi-class adaptive thresholds achieved improvements of 0.1% mAP for cars, 1.1% mAP for pedestrians and 1.9% mAP for cyclists. Compared to fixed matching thresholds, the effectiveness and superiority of the multi-class adaptive dynamic threshold strategy are evident. The primary reason is that the detection capability of the teacher network continuously improves through iterations, and the adaptive dynamic thresholds accommodate this important characteristic.

### 3.5. Qualitative Results and Analysis

**Figure 8:**
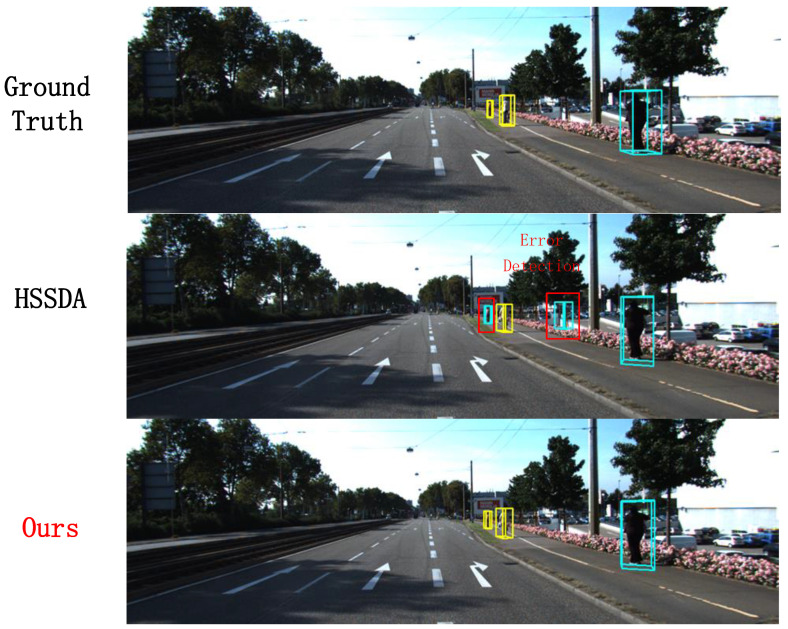
Comparison of cyclist detection results.

**Figure 9:**
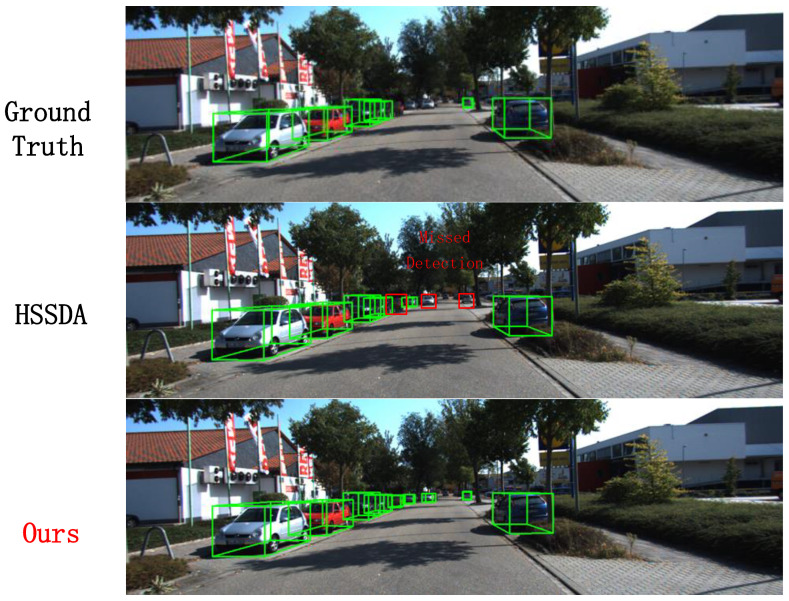
Comparison of car detection results.

**Figure 10:**
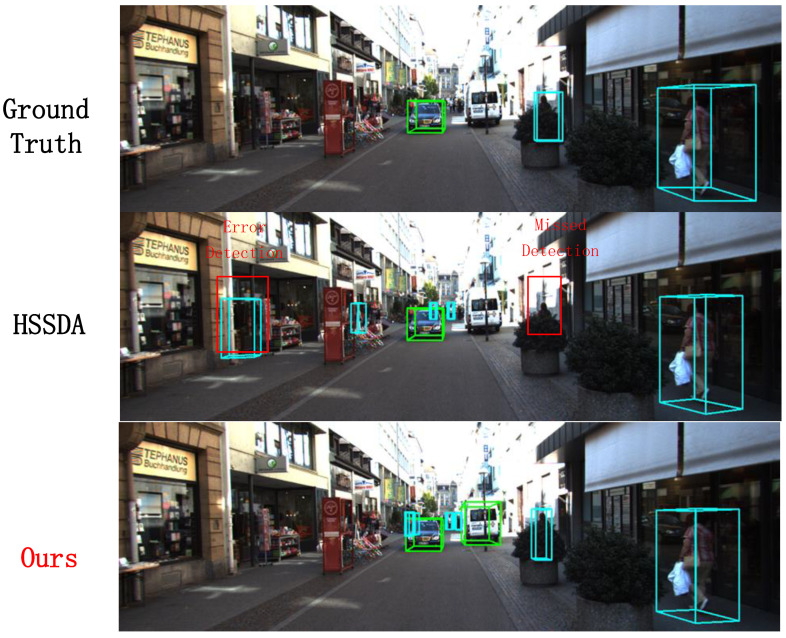
Comparison of pedestrian detection results.

In this section, we visualize the results of the proposed method and compare them with the baseline model and ground truth boxes. The 3D detection results from the point cloud are projected onto 2D images for a more intuitive display. And the reasons for the superior performance of the 1% labeled data are also discussed. In Figure 8, our method successfully detects a pedestrian that the baseline model missed, as well as correcting the false detection of a distant small cyclist target. In Figure 9, our method not only successfully detects the vehicles identified by the baseline model but also accurately detects multiple distant small vehicle targets.In Figure 10, our method successfully detects a pedestrian missed by the baseline model. These visualizations demonstrate that, besides performing well on conventional and short-range targets, our proposed method also enhances detection performance for distant small targets. This is achieved by using multi-modal information as supplementary data and employing a multi-class adaptive threshold strategy to generate dynamic thresholds.

For data with different labeling proportions, the teacher network generates new pseudo-labels during each training session, refreshing the previous pseudo-labels, which are then used as labeled data in the next round of training. Upon completing the second-to-last round of training, the teacher network performs a final inference on all unlabeled scenes and generates pseudo-labels. PE-MCAT evaluates the extraction accuracy of strong pseudo-labels for the three categories, cars, pedestrians, and cyclists, with the results shown in Table 6. The numbers in the table represent the total number of strong pseudo-labels, as well as the total number of labeled instances in all scenes after removing the labeled scenes. It can be observed that after training with only 1% of the labeled data, the final number of extracted strong pseudo-labels has already exceeded 2%. This is because when using 1% labeled data, there is more unlabeled data compared to using 2% labeled data. The large quantity of high-quality strong pseudo-labels, filtered through the PE module and MCAT strategy, explains why the detection results using 1% labeled data are still outstanding. Furthermore, since the pre-trained model is trained based on labeled data, even though the detection capability of the network model trained with only 1% labeled data is close to that of the baseline model HSSDA trained with 2% labeled data, there is still a certain gap compared to the PE-MCAT model trained with 2% labeled data.

Although the proposed point enhancement module and the novel boundary threshold generation strategy demonstrate significant advantages in improving pseudo-label quality and network generalization capability, there are still some limitations. First, while the multi-feature fusion and adaptive threshold strategies can effectively enhance the accuracy of pseudo-labels, their robustness in highly complex or dynamic scenarios has not been fully validated. Second, although the joint variable threshold strategy can generate high-quality pseudo-labels, in extreme cases (such as when labeled data are extremely scarce or data distribution is highly imbalanced), the number of generated pseudo-labels may be insufficient, thereby limiting the learning performance of the student network. Lastly, although these methods perform well on the datasets used, their adaptability to broader application scenarios and other types of datasets still requires further research and validation.

## 4. Conclusions

PE-MCAT proposes a 3D semi-supervised object detection framework, PE-MCAT, based on multi-feature fusion. For the first time in the semi-supervised domain, the framework introduces a data-level multi-feature fusion method, which enhances LiDAR 3D point cloud representations by integrating local and self-features, thereby improving the quality of pseudo-labels. Additionally, the proposed multi-class adaptive threshold strategy dynamically adapts to the evolving detection capabilities of the teacher network, further optimizing the quality of the generated pseudo-labels. Experiments on the KITTI dataset validate the effectiveness of the two components in PE-MCAT. Under the same experimental conditions, PE-MCAT outperforms state-of-the-art methods on both the KITTI and DAIR-V2X datasets with only 2% labeled data, demonstrating its superior performance.

The results of this study indicate that PE-MCAT significantly improves the accuracy of semi-supervised 3D object detection, especially in scenarios with limited labeled data, showing strong generalization capabilities. In future research, we aim to explore the application of PE-MCAT’s multi-class adaptive threshold strategy in other 2D/3D object detection frameworks, especially across different datasets and scenarios. Additionally, further optimization of the feature fusion mechanism and expanding its application to multi-modal data, such as RGB-D and multi-spectral data, will be key areas of future investigation to enhance its adaptability and robustness in more complex environments.

## Figures and Tables

**Table 1:** The performance for cars, pedestrians, and cyclists on the KITTI validation set after training with different ratios of labeled KITTI training set scenes.

Model	Modality	1%				2%			
		Car	Ped.	Cyc.	mAP	Car	Ped.	Cyc.	mAP
PV-RCNN	LiDAR	73.5	28.7	28.4	43.5	76.6	40.8	45.5	54.3
3DIouMatch (PVR.-based)	LiDAR	76.0	31.7	46.4	48.0	78.7	48.2	56.2	61.0
DetMatch (PVR.&FR.-based)	LiDAR + RGB	77.5	57.3	42.3	59.0	78.2	54.1	64.7	65.6
DDS3D (PVR.-based)	LiDAR	76.0	34.8	38.5	49.8	78.9	49.4	53.9	60.7
HSSDA (PVR.-based)	LiDAR	80.9	51.9	45.7	59.5	81.9	58.2	65.8	68.6
Ours (PVR.-based)	LiDAR + RGB	**82.2**	**59.1**	**60.9**	**67.4**	**82.7**	**61.9**	**68.8**	**71.1**

Note: Bold values in the table indicate the best performance.

**Table 2:** The performance for cars, pedestrians, and cyclists on the DAIR-V2X validation set.

Model	Car			Ped.			Cyc.			mAP
	Easy	Mod	Hard	Easy	Mod	Hard	Easy	Mod	Hard	-
SA-SSD	47.76	38.18	36.47	24.245	22.85	22.97	17.10	18.11	17.04	27.19
PV-RCNN	50.43	38.95	36.78	28.67	25.76	24.35	26.57	24.80	23.55	31.10
HSSDA (baseline)	56.02	**46.17**	42.27	34.87	38.10	38.83	37.41	34.38	32.28	40.04
PE-MCAT (ours)	**56.58**	46.05	**43.05**	**47.99**	**50.25**	**51.88**	**51.80**	**48.63**	**41.77**	48.66
Delta	+0.56	−0.12	+0.78	+13.12	+12.15	+13.05	+14.39	+14.25	+9.49	+8.63

Note: Bold values in the table indicate the best performance.

**Table 3:** The ablation of the multi-feature fusion in the point enrichment module.

KITTI	Semantic Information	Manhattan Distance	Relative Position	Car	Ped.	Cyc.	mAP
✔				81.9	58.2	65.8	68.6
✔	✔			82.2	58.5	66.7	69.2
✔	✔	✔		82.2	59.1	66.9	69.4
✔	✔	✔	✔	**82.4**	**59.5**	**67.2**	**69.7**

Note: Bold values in the table indicate the best performance.

**Table 4:** Performance comparison between HSSDA and MCAT models with and without PE module.

Model	Dimension	Car	Ped.	Cyc.	mAP
HSSDA	-	81.9	58.2	65.8	68.6
	PE Module	82.4	59.5	67.2	69.7
MCAT	-	82.3	59.5	67.6	69.8
	PE Module	**82.7**	**61.9**	**67.8**	**71.1**

Note: Bold values in the table indicate the best performance.

**Table 5:** The ablation of the range of the multi-class adaptive threshold.

Threshold	Car	Ped.	Cyc.	mAP
0.3	82.2	57.9	65.6	68.5
0.4	82.0	58.1	65.5	68.4
0.5	81.9	58.2	65.8	68.6
0.6	81.5	58.4	65.3	68.4
Ours	**82.3**	**59.5**	**67.6**	**69.8**

Note: Bold values in the table indicate the best performance.

**Table 6:** Evaluation of strong pseudo-label extraction under different labeling proportions.

Category	1%	2%
Car	6173/14,046	5458/14,046
Pedestrian	426/2152	409/2152
Cyclist	167/720	243/720

## Data Availability

The original data presented in the study are openly available in https://www.cvlibs.net/datasets/kitti and https://thudair.baai.ac.cn/index (accessed on 27 October 2024).
